# Olfactory bulbectomy induces neurobiological alterations in the prefrontal cortex and hyperlocomotion in male rats

**DOI:** 10.1371/journal.pone.0339028

**Published:** 2026-01-27

**Authors:** Mario Alberto Bautista-Carro, Patricia Sánchez-Teoyotl, Daniel Juárez-Serrano, Tommaso Iannitti, Alfonso Díaz, Gonzalo Flores, Julio César Morales-Medina

**Affiliations:** 1 Centro de Investigación y de Estudios Avanzados del Instituto Politécnico Nacional, Unidad Tlaxcala, Tlaxcala de Xicohténcatl, México; 2 Departamento de Fisiología, Biofísica y Neurociencias, Centro de Investigación y de Estudios Avanzados del Instituto Politécnico Nacional, Ciudad de México, México; 3 Facultad de Ciencias Químicas, Benemérita Universidad Autónoma de Puebla, Puebla, México; 4 Department of Medical Sciences, Section of Experimental Medicine, University of Ferrara, Ferrara, Italy; 5 Instituto de Fisiología, Benemérita Universidad Autónoma de Puebla, Puebla, México; Suez Canal University Faculty of Medicine, EGYPT

## Abstract

Major depressive disorder (MDD) is a leading cause of disability and encompasses various subtypes, including agitated depression which is associated with psychomotor agitation and elevated suicide risk. Although the olfactory bulbectomy (OBX) model has been extensively utilized to study depression-related behaviors, most studies have focused on the hippocampus, leaving the role of the prefrontal cortex (PFC) less explored. In this study, we examined the behavioral responses to novelty in the open field test and examined glial and neuronal alterations in the PFC of OBX rats. Our findings revealed that OBX induced hyperlocomotion, consistent with agitated depression. At the cellular level, OBX selectively increased the number of glial fibrillary acidic protein (GFAP)-positive astrocytes in the PFC. These modifications were accompanied by elevated nitric oxide (NO) levels, enhanced c-Fos expression, and a reduction in pyramidal neuron spine density. These findings represent the first integrated evidence of concurrent glial proliferation, NO dysregulation, and impaired neuronal plasticity in the PFC following OBX. Collectively, our results highlight the translational relevance of OBX as a model of agitated depression and point to astrocytic dysfunction and glial-neuronal interaction in the PFC as key contributors to synaptic and behavioral abnormalities in MDD.

## Introduction

Major depressive disorder (MDD) is a leading disability worldwide, with numerous symptoms and varied presentations across individuals [1]. Since 2019, this disorder has been considered a leading cause of disability worldwide and is expected to become the leading cause of disease burden globally by 2030 [2]. Moreover, the 12-month prevalence of MDD is approximately 6% across various countries, while its lifetime risk is substantially higher, ranging from 15% to 18% [3]. There are different MDD subtypes, including agitated depression, where the patient displays hyperlocomotion, for review see Alhau et al. [4]. There are numerous hypotheses regarding the underlying mechanism of MDD, and among those, the latest is the neuro-immunological. This hypothesis suggests that dysregulation of the immune system plays a central role in the development and maintenance of MDD [5]. Glia constitute the largest percent of cells within the central nervous system (CNS), with microglia and astrocytes considered the most important glial cells [5,6]. They give support to neurons secreting numerous molecules including nitric oxide (NO). Moreover, cumulative evidence suggests major glial alterations in the prefrontal cortex (PFC) and hippocampus [7]. In tissues from MDD patients, modifications in proportion and transcriptional profiles of microglia [8] and reductions in astroglia density and size have been observed [7] in the PFC in addition to increased glial density in the hippocampus [9]. Therefore, these results support the neuro-immunological hypothesis of MDD.

Recent evidence suggests that there is an association between olfaction and MDD [10,11]. The olfactory bulbectomy (OBX) has been proposed as an animal model of depression-related behavior. A core feature of this model is hyperlocomotion in a novel environment, a trait interpreted as a failure to adapt to a novel environment [12,13]. Previously, our group observed reduced cell proliferation in the dentate gyrus of the dorsal hippocampus and dendritic rearrangement in CA1 pyramidal neurons [14]. In the CA1, an increase in processes and number of glial fibrillary acidic protein (GFAP)-positive astrocytes and a reduction of processes in Ionized Calcium-Binding Adapter Molecule 1 (Iba1)-positive microglia have been observed [15]. Moreover, there is a key connection between the hippocampus and the PFC [16,17]. These results suggest a high incidence of glial alteration in the OBX rat. However, studies in this model of depression-related behavior have focused on the hippocampus, for review see [13]. Little is known regarding neuronal and glial alterations in the PFC.

MDD, particularly its agitated subtype, remains a highly disabling condition with limited mechanistic understanding. OBX model has long been used to mimic behavioral and neurochemical alterations associated with depression, yet most studies have focused on the hippocampus or global cortical changes. Given the increasing recognition of the PFC as a critical hub for cognitive and affective regulation, clarifying its role in OBX-induced pathology is essential. Therefore, the expression of Iba1-positive microglia and GFAP-positive astrocytes, c-Fos positive cells, the morphology and spine density of pyramidal neurons, and NO levels in the PFC will be investigated, along with behavioral testing in the open field test (OFT) in the OBX rats.

## Results

### Study design

**Fig 1:**
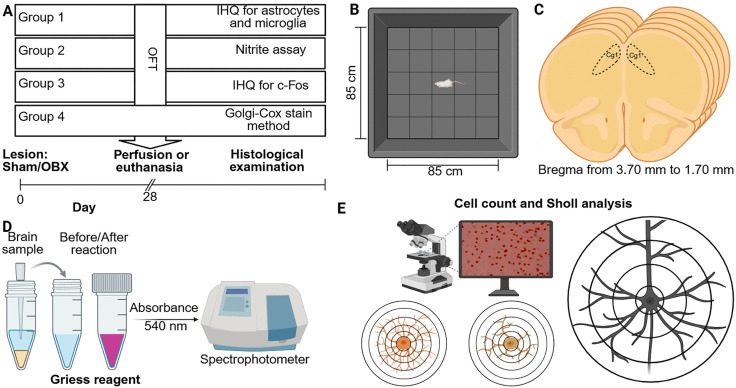
Experimental design. **(A)** Representative diagram of each experimental group. OFT was performed at 28 days post-OBX (except for group 4), then perfusion or euthanasia was performed for the corresponding histological examination. **(B)** Representative image of OFT. **(C)** Figurative image of coronal slices of the PFC at +3.70 mm and +1.70 mm from bregma. **(D)** Nitrite assay by Griess method. **(E)** Cell counts were performed at each labeling and Sholl analysis for astrocytes, microglia and neurons. Also, determination of the density of dendritic spines to pyramidal neurons was performed. *Abbreviations. OFT, open field test; OBX, olfactory bulbectomy; IHQ, immunohistochemistry; PFC, prefrontal cortex.* Created in BioRender. Morales Medina, J. (2025) https://BioRender.com/n50l212.

To test the consequences of OBX, four groups of rats were included in our experiments (Fig 1).

### OBX causes hyperactivity in the OFT

**Fig 2:**
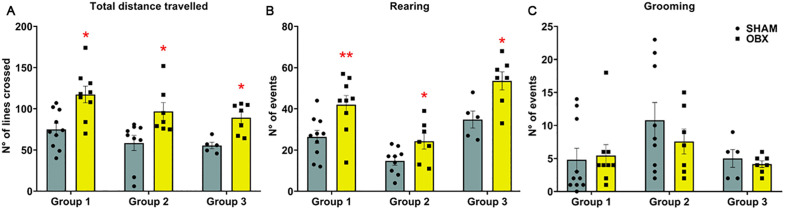
OBX causes hyperlocomotion in the OFT. OBX increases total locomotion **(A)** and frequency of rearing **(B)** but not grooming behaviors **(C)**. These results correspond to groups 1-3. Data are mean + /-S.E.M. with n = 9-10, 7-9 and 5–7 rats per group, respectively. *< p 0.05, **≤ p 0.01 compared to control group. *Abbreviations. OFT, open field test; OBX, olfactory bulbectomy; N°, number.*

As anticipated, OBX increased distance traveled (unpaired t-test *t*(17) = −3.466, p = 0.0030; *t*(14) = −2.732, p = 0.0162; and *t*(10) = −3.763, p = 0.0037; Fig 2A, for groups 1–3, respectively) and frequency of rearing (unpaired t-test *t*(17) = −2.900, p = 0.0100; *t*(14) = −2.311, p = 0.0366 and *t*(10) = −2.984, p = 0.0137; Fig 2B, for groups 1–3, respectively) in the OFT. In the same test, OBX did not modify grooming (Fig 2C, for groups 1–3).

### OBX increases astrocyte density without altering the length or arborization of their processes in the PFC

**Fig 3:**
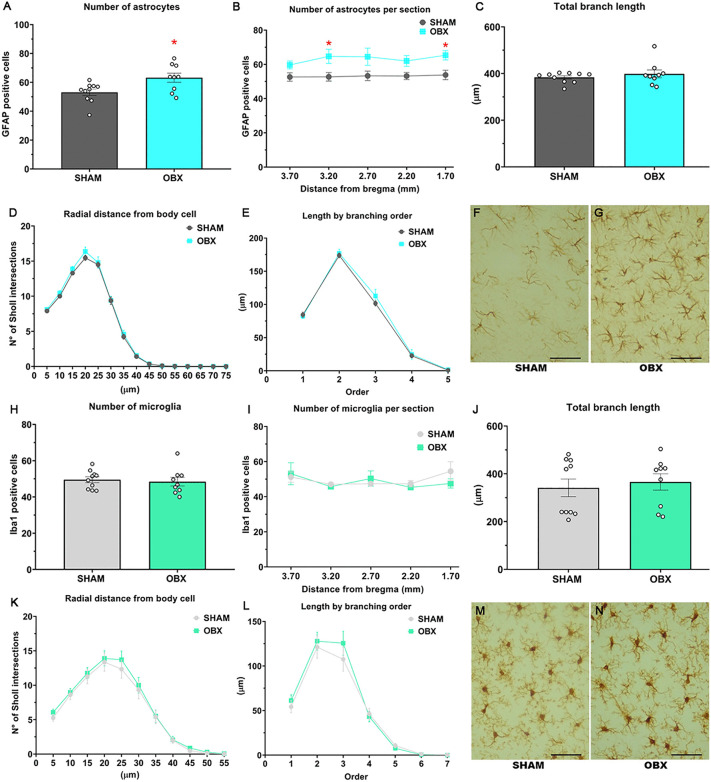
OBX causes glial alterations in the PFC. **(A)** The total density of astrocytes is increased in the OBX group. **(B)** Increased astrocyte numbers were observed at very specific locations in relation to bregma in the OBX group. Sholl analysis revealed no differences in astrocyte morphology (**C**, **D** and **E**). **(H)** Microglial density was unchanged both overall (**I**) and at specific locations from bregma. Sholl analysis also revealed no differences in microglial morphology (**J**, **K** and **L**). Representative photomicrographs of astrocytes and microglia are shown in (**F**, **G**, **M** and **N**), respectively. The figures were taken at 400X magnification, and the scale is equal to 50 µm. These results correspond to group 1. Data are mean + /-S.E.M. with n = 9-10 rats per group. * p < 0.05 compared to control group. *Abbreviations. PFC, prefrontal cortex; OBX, olfactory bulbectomy.*

OBX increased the number of GFAP-positive astrocytes (unpaired t-test *t*(17) = −2.678, p = 0.0159, Fig 3A). There is a group effect, but no effect of distance from Bregma [two-way ANOVA, group F (1, 85) = 27.05, p < 0.0001; distance F (4, 85) = 0.3807, p = 0.8219; Interaction F (4, 85) = 0.2322, p = 0.9195] (Fig 3B). Sidak’s *post hoc* analysis revealed significant statistical differences at 3.20 mm and 1.70 mm from Bregma (p = 0.0353 and 0.0464, respectively) compared to Sham-OBX (Fig 3B). Sholl’s analysis revealed no differences in total branching length (Fig 3C), arborization of astrocyte branches (Fig 3D) or length of each branch order (Fig 3E). Representative photomicrographs of GFAP-labeled astrocytes are shown in Fig 3F and Fig 3G. The figures were taken at 400X magnification, and the scale is equal to 50 µm.

### OBX does not alter the density or morphology of microglia in the PFC

Iba-1 positive microglial density was unchanged overall (Fig 3H) and at specific locations from bregma in OBX animals (Fig 3I). Sholl’s analysis revealed no differences in total branching length (Fig 3J), arborization of microglia branches (Fig 3K) and length for each branch order (Fig 3L). Representative photomicrographs of Iba1-positive microglia are shown in Fig 3M and Fig 3N. The figures were taken at 400X magnification, and the scale is equal to 50 µm.

### OBX does not alter the density or morphology of astrocytes and microglia in the CA1

**Fig 4:**
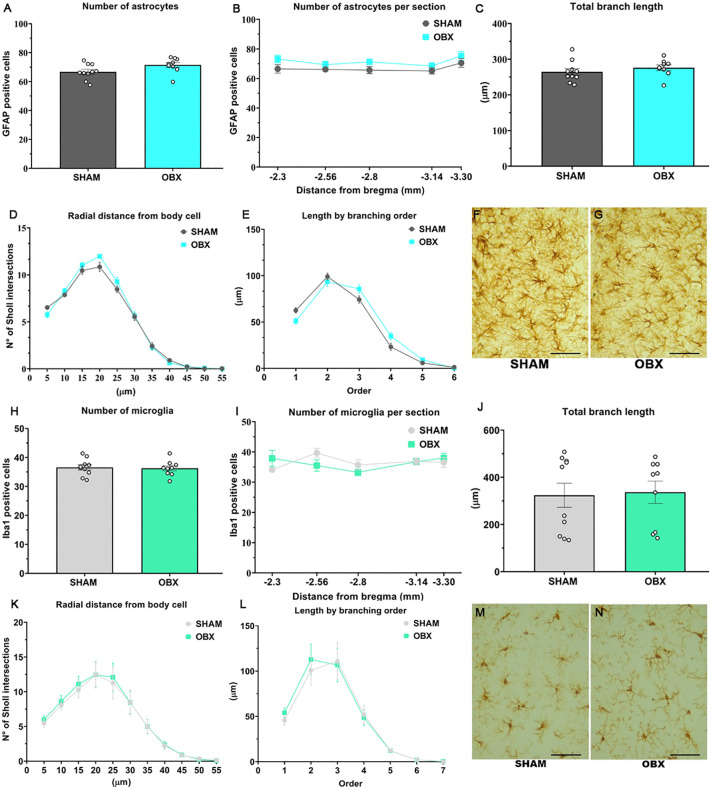
OBX does not cause glial alterations in the CA1 of the dorsal hippocampus. **(A** and **H)** Astrocyte and microglial density were not modified by OBX. (**B** and **I**) Glial quantification per section at specific locations from bregma were not altered. Sholl analysis revealed no differences in astrocyte (**C**, **D** and **E**) or microglia morphology (**J**, **K** and **L**). Representative photomicrographs of astrocytes and microglia are shown in (**F**, **G**, **M** and **N**), respectively. The figures were taken at 400X magnification, and the scale is equal to 50 µm. These results correspond to group 1. Data are mean + /-S.E.M. with n = 9-10 rats per group. * p ≤ 0.05 compared to control group. *Abbreviations. OBX, olfactory bulbectomy; CA1, cornus Ammon 1.*

OBX did not modify CA1 astrocyte or microglia density (Fig 4A and Fig 4H) regardless of distance from the bregma (Fig 4B and Fig 4I). Sholl analysis revealed no differences in total branching length (Fig 4C and Fig 4J), arborization of branches (Fig 4D and Fig 4K), or length for each branching order (Fig 4E and Fig 4L). Representative microphotographs of astrocytes (Fig 4F and Fig 4G) and microglia (Fig 4M and Fig 4N). The figures were taken at 400X magnification, and the scale is equal to 50 µm.

### OBX increases NO levels in the PFC

**Fig 5:**
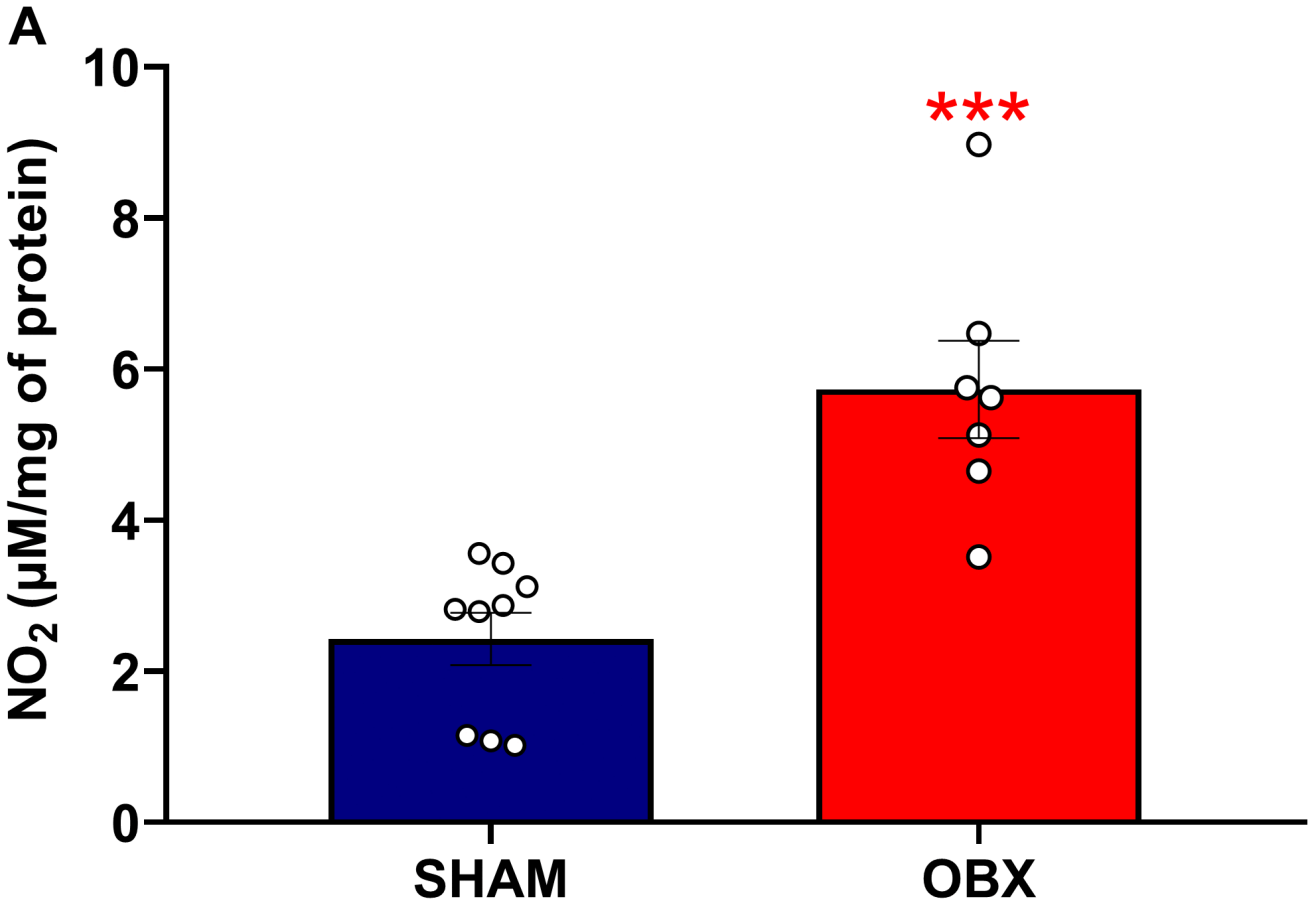
OBX increases NO production in the PFC. After completion of the behavioral test, we identified an increase of NO_2_ in the OBX group **(A)**. The values are the mean of nitrites μM/mg of protein. These results correspond to group 2. Data are mean + /-S.E.M. with n = 7-9 rats per group. ***p ≤ 0.001 compared to control group. *Abbreviations. OBX, olfactory bulbectomy; NO, nitric oxide; PFC, prefrontal cortex.*

NO release was assessed by measuring its stable metabolite NO_2_. We found an increase of NO_2_ in the OBX group (unpaired t-test *t*(14) = −4.790, p = 0.0003, Fig 5A).

### OBX increases the number of c-Fos-positive cells in the PFC

**Fig 6:**
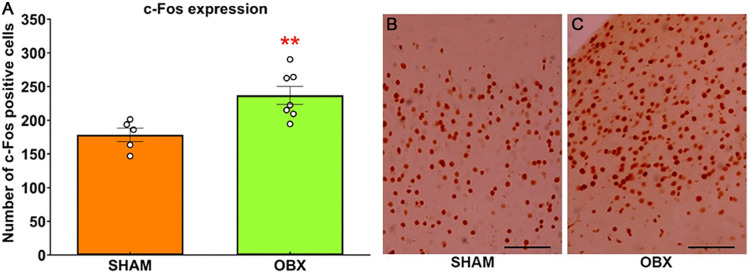
OBX increases c-Fos expression in the PFC. **(A)** Histological examination showed increased c-Fos expression in the PFC in the OBX model. **(B** and **C)** The photomicrographs were taken at 400X magnification, and the scale is equal to 50 µm. These results correspond to group 3. Data are mean + /-S.E.M. with n = 5–7 rats per group. **p ≤ 0.01 compared to control group. *Abbreviations. OBX, olfactory bulbectomy; PFC, prefrontal cortex.*

The number of c-Fos-positive cells was higher in rats with OBX surgery (unpaired t-test *t*(10) = −3.250, p = 0.0087, Fig 6A). Representative photomicrographs of c-Fos positive cells (Fig 6B and Fig 6C). The figures were taken at 400X magnification, and the scale is equal to 50 µm.

### OBX decreases dendritic spine density in the PFC

**Fig 7:**
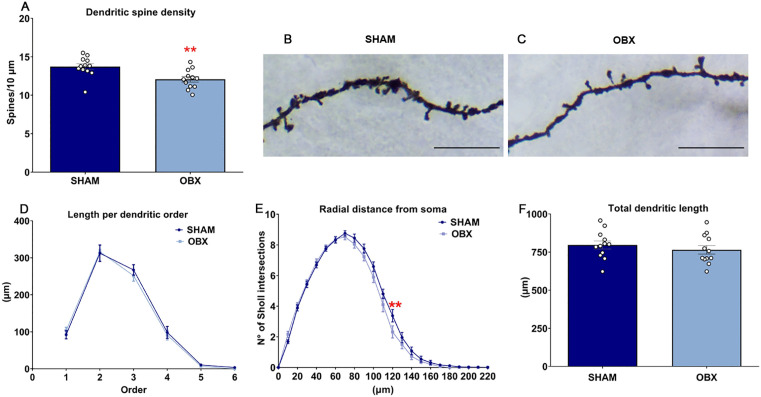
OBX decreases the density of dendritic spines and modifies the arborization of pyramidal neurons of the PFC. **(A)** OBX decreases the dendritic spine density. **(B** and **C)** Representative photomicrographs of a Golgi-stained distal dendritic segment. **(D)** The length of each dendritic order was not modified by OBX surgery. **(E)** Radial arborization was modified 120 µm from the soma of the pyramidal neurons. **(F)** OBX does not alter total dendritic length. The photomicrographs were taken at 2000X magnification, and the scale is equal to 10 µm. These results correspond to group 4. Data are mean + /-S.E.M. with n = 12 rats per group. **p < 0.01 compared to control group. *Abbreviations. OBX, olfactory bulbectomy; PFC, prefrontal cortex; N°, number.*

The density of spines in distal dendritic branches was significantly lower in the OBX group compared to controls (unpaired t-test *t*(22) = 3.136, p = 0.0048, Fig 7A). Sholl’s analysis showed specific differences in the arborization of dendrites [two-way ANOVA, group F (1, 506) = 7.284, p = 0.0072; intersection F (22, 506) = 508.2, p < 0.0001; Interaction F (22, 506) = 1.363, p = 0.1259] (Fig 7E). Sidak’s *post hoc* analysis revealed a significant statistical difference from Sham-OBX in the dendritic spine density 120 µm from bregma (p = 0.0093; Fig 7E). Sholl analysis revealed no differences in length for each dendritic order (Fig 7D) or total length of the dendrites (Fig 7F). Representative photomicrographs of a Golgi-stained distal dendrite segment are shown in (Fig 7B and 7C).

## Discussion

In this study, we observed that OBX induces hyperlocomotion as previously reported by our group and others [12,13,18], a hallmark of agitated depression. This model also produces a constellation of glial and neuronal changes in the PFC including selective astrocytic proliferation, elevated NO levels, increased neuronal activation and reduced spine density in pyramidal neurons. Moreover, rats with OBX did not change their food intake (S1A-S1D Fig). Altogether, these results highlight the relevance of the PFC in the OBX rat.

### OBX increases hyperlocomotion to novelty

Agitated depression is a subtype of MDD characterized by psychomotor agitation, restlessness and repetitive behaviors associated with higher suicide risk, for review see Alhau et al. [4]. In the present study we observed that OBX increased locomotion and rearing across the groups evaluated. This aberrant behavior has been previously observed in rats and mice of different strains and interpreted as a failure to adapt to novel environment and to evaluate risk assessment [12–14,19]. In normal conditions, rearing is considered an exploratory behavior, but when it is excessive and seemingly purposeless it can be considered repetitive behavior [20]. Given the robustness of hyperlocomotion as well as other behaviors measured in this animal model including rearing, OBX is often referred as a model of agitated depression.

### OBX increases the number of GFAP-positive astrocytes in the PFC

A strong association between MDD and the hippocampus has been previously observed [9,21]. In addition, the PFC is implicated in numerous physiological processes including working memory and attention, and more importantly it integrates hippocampal information to guide future actions [22,23]. Therefore, the role of the PFC in MDD is increasing.

Glial cells are the most abundant cell type in the CNS [24]. Notably, astrocytes and microglia have been implicated in the pathophysiology of MDD [24–26]. Astrocytes are the most abundant glial cells in the CNS and play a crucial role in synaptic function, providing metabolic substrates and cytokine release [3,27,28]. Additionally, microglia, which constitute 10–15% of CNS cells, serve as the resident immune cells and regulate neuroinflammatory response [29]. Under pathological conditions, glial cells proliferate and undergo morphological changes indicative of activation; astrocytes extend and elongate their processes, while microglia enlarge their cell bodies and retract their processes [3,29]. Emerging evidence has suggested a role of glial alterations, particularly in the PFC in MDD [7,25,30–34].

In the present study, we observed a selective increase in GFAP-positive astrocytes in the PFC in the OBX rat. Previously, Machado et al. [19] found an increase in GFAP expression in the cortex in this model, however, western blot data could not distinguish between a modification in density or morphology. In a later study, an increase was also observed in a receptor found in astrocytes, the adenosine A1 receptor. Moreover, our group observed that OBX led to an increase in both the complexity of processes and density of GFAP-positive astrocytes in the hippocampus (CA1), while concurrently reducing the morphological complexity of Iba1-positive microglia in the same hippocampal subfield and in the PFC [15]. While the glial rearrangements are measured four weeks after the lesion, there are clear methodological differences between both studies. In the previous study, animals were placed individually in Plexiglas cages for behavioral assessment once a week [15]. Meanwhile, in the present study animals were mostly undisturbed after the surgery. The OBX rat is known to display increased levels of corticosterone (CORT) [35]. Housing rats individually in cages that restrict their movement is comparable to 10mG/Kg CORT [36] and therefore we observed the result of the lesion and the spikes of CORT, also known to activate glial cells. Moreover, chronic stress, an animal model of depression related behavior, induced a reduction in astrocytic processes in the PFC in mice [37] and rats [38]. In this study we observed PFC astrocytic alterations induced only after OBX, similar to other models of depression-related behavior and MDD, which strengthens the translational relevance of this model.

### OBX increases NO levels in the PFC

NO functions as a critical intercellular messenger in the CNS [39,40]. While physiological NO concentrations participate in processes such as vasodilation at the vascular level and in long-term potentiation at the neuronal level, dysregulated levels of NO and nitrosative species may contribute to pathological conditions [39,40]. NO is synthesized by three nitric oxide synthases: inducible (iNOS), endothelial (eNOS) and neuronal (nNOS) [39]. Both astrocytes and microglia contain iNOS and upon appropriate stimulation, can increase NO production [41,42]. In the current study, we observed that OBX increased this intercellular messenger in the PFC. In apparent agreement, OBX elevated NO levels in the cerebral cortex [43]. Furthermore, Ploska et al. [44] observed that in this animal model, eNOS and nNOS expression in the PFC remains unaltered. Collectively, these findings suggest that the observed increase in NO is iNOS-dependent. This abnormal elevation in NO may be attributed to the proliferation of astrocytes in this brain region. Moreover, prenatal lipopolysaccharide infection resulted in hyperlocomotion and increased NO levels in the PFC in rats [45], suggesting an association between altered locomotion and NO levels in the cortex. This NO increase in the PFC and hyperlocomotion was also observed in the current study.

### OBX decreases spine density in PFC neurons

Neuronal plasticity includes alterations in the dendritic arbor and spines due to internal or external stimuli [6,46]. In the present study, we observed decreased spine density in PFC neurons. The abundance of SNAP-25, a protein involved in synaptic vesicle fusion and neurotransmitter release, is reduced in the frontal cortex in this animal model [19]. In apparent agreement, synaptic loss has been observed in the PFC of patients with MDD [47]. These modifications in plasticity might be attributed to the modifications observed here in astrocytes, since these cells (part of the quapartite synapsis) could impact neuronal excitability [28,48]. The proliferation of astrocytes in this brain region, together with altered NO levels, may contribute, at least in part, to the observed modifications in spine density. Additionally, the metabolism of arachidonic acid, particularly the synthesis of epoxyeicosatrienoic acids (EET), occurs within astrocytes; Notably, ETT levels are reduced in an animal model of depression [49], further suggesting a role of astrocytes in the PFC in this disorder. Further, astrocytic-induced hypometabolism in CORT-treated rats, a model of depression, was observed in this brain region [50]. Moreover, in mice subjected to social defeat, another model of depression-related behavior, lower levels of astrocytic-related ATP were observed [51]. These results further suggest that selective glial alterations in the PFC induce changes in neuroplasticity.

### OBX increases the activity of cells in the PFC

c-Fos is an immediate-early gene commonly utilized as a molecular marker for mapping neuronal activation in response to various stimuli, including novel experiences [52]. In this study, we observed that OBX increased the number of c-Fos positive cells in the PFC when rats were presented novel stimuli. In clear contrast, Takahashi et al. [53] failed to observe c-Fos alterations in this brain region in the same animal model. However, there are major methodological differences. For example, in the latter case there was no stimulation and the study was conducted in mice. A reduction in astrocyte-related ATP reduce the stimulation of P2X2Rs, a nucleotide receptor from the P2X family, in GABAergic interneurons in the PFC and therefore reduces GABAergic inhibition in an animal model of depression [3]. Therefore, the astrocytic alterations could lead to reductions in GABAergic inhibition and therefore an increase in cellular activity in the PFC.

## Limitations and clinical implications

Only male rats were evaluated, precluding conclusions on sex-related effects that are increasingly recognized in depression research. The glial assessment was limited to the PFC and CA1 of hippocampus, and while these regions are central for integrating emotional and cognitive processes, interaction with other brain areas were not explored here.

The results suggest that astrocytic dysfunction in the PFC contributes to the synaptic and behavioral alterations associated with agitated depression, a severe form of MDD. The observed increase in GFAP-positive astrocytes and NO dysregulation highlights glial cells as potential therapeutic targets. Intervention aimed at modulating astrocytic reactivity, regulating NO signaling, or restoring synaptic plasticity in the PFC may represent novel strategies for treatment resistant or agitated forms of MDD. Furthermore, identifying glial-neuronal interactions as a core pathological feature underscores the need for biomarkers beyond monoaminergic imbalance, potentially guiding the development of precision therapies tailored to specific MDD subtypes.

## Conclusions

These results provide the first integrated evidence that OBX induces concurrent behavioral, glial and neuronal alterations in the PFC. The novelty of this work is related to the association between astrocytic proliferation, NO dysregulation and impaired neuronal plasticity in this brain region. This study therefore advances the translational relevance of OBX as a model of agitated depression and highlights the PFC as a key site where glial-neuronal interactions may contribute to the pathophysiology of MDD.

## Materials and methods

### Animals

Throughout the investigation, 75 adults male Wistar rats (*Rattus norvegicus*) between the ages of two and three months were employed. Rats were obtained from the Centro de Investigación in Reproducción Animal, which is a division of Cinvestav, México. Three to four rats were housed per cage, kept on a 12-hour light/dark cycle with a temperature of 20°C + /- 2°C, and given unlimited access to food and water. Animals were randomly assigned to one of two surgical groups, sham or OBX lesions, and then received post-surgical care. All procedures described in this study complied with the guidelines of CIRA Animal Care Committee (BUI-LT02–2023) and “Norma Oficial Mexicana 062”, the Guide for the Care and Use of Laboratory Animals, and the ARRIVE guidelines [54]. Every attempt was made to reduce suffering of the animals. Before or during the experiments, animals that displayed signs of suffering were promptly examined by a veterinarian and, if required, euthanized.

### OBX surgery

OBX was performed according to previous reports [15,55]. Rats were anesthetized with a ketamine/xylazine cocktail (0.75 ml ketamine + 0.25 ml xylazine + 5 ml sterile saline) and administered intraperitoneally at a dose of 0.125 ml/20 g body weight. Once anesthetized, the head was shaved and asepsis was attained using benzalkonium chloride and iodopovidone, then an incision was made on the midline of the skull from the forehead to the dorsum of the nose (1 cm). Finally, the aponeurosis and periosteum were uncovered to reach the bone. Two holes of 2 mm diameter were drilled with a microdrill, located 8 mm anterior to bregma and 2 mm from the midline of the frontal bone. The olfactory bulbs were aspirated bilaterally using a cannula connected to a distilled water pump, taking care not to cause damage to the PFC. Sham rats were operated in a similar manner, except that olfactory bulbs were not removed. To prevent blood loss, the orifices were filled with hemostatic sponge (Spongostan, Ethicon, Inc. USA), sutured continuously and covered with iodopovidone. Finally, 4 ml of Hartmann’s solution (PiSA, México) was administered subcutaneously. Animals remained in pairs in their respective cages for four weeks to recover. The analysis only included data from animals whose olfactory bulbs had been completely removed and whose frontal cortex had not been damaged, as assessed by examination after brain removal. In total, 4 OBX rats were excluded from the present study. For more details, see (dx.doi.org/10.17504/protocols.io.81wgbwqjngpk/v1).

### Open field test

This test was performed in an open black square arena (85 x 85 x 60 cm). The field was divided into 25 equally sized squares, with 300 LUX of illumination [55,56]. After 1 h of habituation in the laboratory, the rat was placed in the center of the field and allowed to explore for 5 min. The rat was then removed from the field and placed in a new box separate from its group. After each trial, the box was cleaned at the base and walls with 70% alcohol to eliminate olfactory cues. During the 5 minute exploratory period, the experimenter left the laboratory. To control for possible circadian cycle effects, all tests were performed at the same time of day between 7:00 am and 12:00 pm. All tests were videotaped with a central view of the field at a height of 2 m to the ground. The video camera used was a Canon (model Vixia HF R70) with a 2.8–89.6 mm lens. An observer blind to the injury recorded locomotion and frequency of rearing and grooming. For more details, see (dx.doi.org/10.17504/protocols.io.yxmvmbqo5g3p/v1).

### Immunohistochemistry

At the end of OFT, group 1 was perfused immediately and group 3 two hours after the test. For group 3 (c-Fos), perfusion was delayed 2 hours in to allow for peak c-Fos expression after the test [57]. Rats were deeply anesthetized with a ketamine/xylazine cocktail and perfused transcardially with 300 ml of fresh cold phosphate buffered saline (1x PBS) (pH 7.4), followed by 300 ml of 4% paraformaldehyde in 1x PBS as previously reported [15,52]. Brains were removed and postfixed in 4% paraformaldehyde for 48h. Brains were cut at 40μm width on a vibratome (Leica, VT1000S microsystem, USA). Coronal slices were made using the coordinates of Paxinos and Watson [58] of the PFC (+3.70 to +1.70 mm bregma) and CA1 of the dorsal hippocampus (−2.3 to −3.30 mm bregma). Slices were collected sequentially in four-row wells coated with 1x PBS. After sectioning all brains, 1x PBS was removed and the sections were placed in cryoprotectant [glycerol: ethylene glycol: PBS 1x, (3:3:4)] and stored at −20 °C until use. For more details, see (dx.doi.org/10.17504/protocols.io.3byl46de8go5/v1).

#### Group 1.

On day 1, the sections were washed in 1x PBS 4 times for 5 minutes each. Subsequently, the sections were incubated in 1.5% hydrogen peroxide (H_2_O_2_) dissolved in 1x PBS for 5 minutes. Afterwards, the sections were rinsed in 1x PBS 2 times for 5 minutes each. Next, the sections were incubated with the blocking solution [1x PBS + 0.3% Triton X – 100 + 3% normal sera (rabbit for GFAP and goat for Iba-1)] for 2 hours. Finally, we incubated for 24 hours at room temperature in primary antibody (Ab) cocktail consisting of [goat anti-GFAP (Abcam #ab53554, 1:500) or rabbit anti-Iba-1 (Cell Signaling, 1:1000) in diluent antiserum (1x PBS + 0.3% Triton X – 100 + 1% normal sera (as indicated above). On day 2, sections were washed in 1x PBS 3 times for 5 minutes each. Subsequently, sections were incubated for 1 hour at room temperature using anti-Goat IgG, VECTASTAIN, Elite ABC-HRP Kit (1:250, Vector Labs #PK-6105, CA, USA) for GFAP or anti-Rabbit IgG, VECTASTAIN, Elite ABC-HRP Kit (1:500, Vector Labs #PK-6101, CA, USA) for Iba1. After incubation the sections were washed 3 times in 1x PBS for 5 minutes each. Subsequently, the sections were incubated in avidin-biotin complex (ABC) for 1 hour at room temperature. The sections were washed 3 times in 1x PBS for 5 minutes each. The sections were then incubated with 3–3’-diaminobenzidine (DAB) for 2 minutes 30 seconds (Peroxidase substrate kit DAB, SK-4100, Vector Laboratories Inc., CA, USA,). Finally, the sections were washed in 1x PBS 2 times for 5 minutes each and 1 time in distilled water for 5 minutes and then mounted on gelatinized slides for subsequent counting and morphological analysis. The three-dimensional reconstruction of five glial cells in each hemisphere was completed by a blinded observer (10 cells per animal, 5 cells per cerebral hemisphere). A camera lucida (DM 2000 Microscope, Leica Microsystems, USA) was used to recreate each cell in two dimensions using a 40X objective lens. The Sholl analysis was used to quantify glial processes [59], modified for glial cells [15]. A transparent grid with concentric rings spaced 5 µm apart was placed on each glial cell. The number of intersections of the rings was quantified. In addition, the total length of the branch was calculated by multiplying the total number of intersections of each ring by 5 µm. The total number of glial branches was counted in each order from the center of the cell body to the end of the glial branch. For more details, see (dx.doi.org/10.17504/protocols.io.3byl46de8go5/v1).

#### Group 3.

*S*ections were washed in 1x PBS 4 times for 5 minutes each, then incubated in 1.5% H_2_O_2_ dissolved in 1x PBS for 5 minutes. The sections were rinsed in 1x PBS 2 times for 5 minutes each. The sections were then incubated in blocking solution (1x PBS + 3% normal rabbit serum + 0.3% Triton X-100) for 120 minutes. Finally, goat anti c-Fos (Abcam #ab156802, 1:500) primary Ab in diluent antiserum (1x PBS + 1% normal rabbit serum + 0.3% Triton X-100) was added to the sections for 24 hours at room temperature. On the next day, sections were washed in 1x PBS 3 times for 5 minutes each. Sections were incubated in secondary Ab using anti-Goat IgG [1:250], VECTASTAIN, Elite ABC-HRP Kit, Peroxidase, Vector Labs #PK-6105, CA, USA) for 1 hour at room temperature. After incubation the sections were washed in 1x PBS 3 times for 5 minutes each. Subsequently, the sections were incubated in ABC for 1 hour at room temperature. The sections were washed in 1x PBS 3 times for 5 minutes each. Then, sections were incubated with DAB for 2.5 minutes (Peroxidase substrate kit DAB, Vector Laboratories Inc. #SK-4100, Burlingame, CA, USA). Lastly, sections were washed in 1x PBS, twice for 5 mins each and 1 time in distilled water for 5 mins and then mounted on gelatinized slides for further count analysis. For more details, see (dx.doi.org/10.17504/protocols.io.3byl46de8go5/v1).

Astrocyte, microglia and positive c-Fos labeling were counted manually at 400X magnification (Leica microscope, model DM200, microsystems, USA). Five coronal sections per brain were counted bilaterally.

### Nitrite assay

#### Group 2.

Animals were decapitated after OFT and the brains were immediately removed. The PFC was dissected as previously reported [60,61]. PFC were homogenized in 3 ml of ice-cold 0.1 M PBS, pH 7.4. The homogenate was centrifuged at 12,500 rpm (4 °C). The supernatant was obtained and stored at −70 °C. NO quantification was performed by Griess method according to previous reports. Griess reagents were sulfanilamide, glacial acetic acid, N-(1-naptyl) ethylenediamine, and sodium nitrite (Sigma, St. Louis, MO, USA). An aliquot of the homogenate (50 μL) was treated directly with 250 μL of the reagent and then incubated for 10 min at 4–8 °C under diminished light. Samples were examined in a spectrophotometer at 540 nm. The protein content of each sample was determined by the Coomassie method [62]. To calculate the data, μM of NO_2_^−^ per milligram (mg) of protein was used. For more details, see (dx.doi.org/10.17504/protocols.io.kqdg31ypel25/v1).

### Golgi-Cox stain

*Group 4*. 28 days post-surgery, the animals were anesthetized with ketamine/xylazine anesthetic cocktail, and perfused intracardially with 0.9% saline solution. Brain tissues were rapidly removed and processed by the modified Golgi-Cox method as described previously [63,64]. Using the coordinates of Paxinos and Watson [58], 200μm thick coronal sections of the PFC [bregma (+3.70 to +1.70)] were obtained on a vibratome (Leica, VT1000S microsystem, USA) and placed on gelatinized slides. Sections were then treated with ammonium hydroxide for 30 min, followed by 30 min in Kodak Film Fixer and finally rinsed with distilled water, dehydrated, and mounted with resinous medium. The neurons were measured using a light microscope (DM 2000 Microscope, Leica Microsystems, USA) equipped with camera lucida. In a two-dimensional plane, ten pyramidal neurons (5 in each hemisphere) from each brain were reconstructed. Dendritic tracing was quantified by Sholl analysis [59]. A transparent grid with concentric rings spaced 10μm apart was placed over the dendritic design, and the number of intersections was estimated. Additionally, the total dendritic length was calculated by multiplying the total number of intersections of each ring by 10μm. Another estimate of dendritic arborization is the total number of dendritic branches that were present in each order of cell body or dendritic shaft. To obtain the density of spines, ten distal and/or last-order dendrites with an approximate length of 10μm (5 for each hemisphere) were drawn at a 1000x resolution. For more details, see (dx.doi.org/10.17504/protocols.io.q26g7n643lwz/v1).

### Statistical analysis

For neurons and glia, branch order length, radial distance and number of cells per section were analyzed by two-way ANOVA, followed by Sidak test for *post hoc* comparisons. OFT, NO, spine densities, total number of astrocytes and microglia, c-Fos, and total branch length were analyzed by a two-tailed unpaired *t*-test. The normality of the data was tested using the Shapiro-Wilk test. p < 0.05 was considered statistically significant. The results were expressed as the mean ± SEM for all experiments. The data was analyzed using GraphPad Prism 8 (GraphPad Software Inc., San Diego, CA, USA).

## Supporting information

S1 Fig Rats with OBX do not change their food intake. (TIF)

S1 Data All raw data. (ZIP)
